# Books

**Published:** 1881-12

**Authors:** 


### Books.

Indigestion, Biliousness and Gout in Its Protean Aspects. Part I. Indigestion and Biliousness. By J. Milner Fothergill, M.D. Pages 320. New York: Wm. Wood & Co. 1881.

This book, the author informs us, is written from a physiological stand-point. The history of normal digestion precedes and introduces the subject of indigestion; first in the alimentary canal, then, secondly in the liver.

Pursuant to the purpose of the writer a concise history of the digestive process is given, and the several digestive fluids and their offices in the economy are distinctly described, according to the latest teaching of modern physiological observers.

All digestion is a process of solution for which previous disintegration is essential, so indigestion may be due to imperfect disintegration or defective solvent power, from impairment of one or more of the digestive fluids. Thus in order to apply the appropriate remedy a correct understanding of the defect must be obtained. This form of dyspepsia is denominated primary; then we have a secondary indigestion, which may be neurosal, reflex, cardiac or toxæmic.

In the chapter devoted to the consideration of diet and drink, the author says: "Strong beef tea, made from almost fabulous amounts of beef, on which the housewife proudly dilates—well, it is the fashion, so it must be spoken with circumspection—is a fraud! or something akin thereto. Its food value is yet to be demonstrated. Not long ago calves-foot jelly was indispensable in conditions of debility; its day is now over, it is little heard of. Strong beef-tea, meat essences, meat juice are pleasant; and certainly patients think they are proper things for the debilitated. Well they may be so; if and provided they take something else as well. But to give the starving patient, starving in the midst of plenty, these things to feed him, is giving him a stone when he asks for bread."

A large part of the book is surrendered to the consideration of questions pertaining to the liver—its functions, and the phenomena of its disturbance—biliousness. The function of the liver is a complex one, and is of great importance, both in the elaboration of the products of digestion and in the destruction by oxidation of waste and surplus albuninoid material, as well as in its secretory action.

"It is the second function of the liver which is so serious a matter in hot climates; and there, as elsewhere, the best way to give physiological rest to a disturbed liver, is to relieve it, as far as possible, from the labor of disintegrating albumonoid matter."

Uric acid is regarded as a product of perverted metabolism in the liver, and urinary deposits are often the result of liver indigestion, even crude albumen may pass unchanged in the liver, thence into the general circulation and be eliminated as albumen by the kidneys—hence imperfect liver digestion is not infrequently mistaken for disordered renal action; and, indeed, the long continued elimination of the products of faulty digestion through the kidneys may result in renal degeneration. The relation between defective liver digestion and diabetes receives attention, and glycosuria is declared to result from the incomplete dehydration of the sugar of the portal vein into glycogen.

Mercury, aloes, podophyllin, rhubarb, jalap, colocynth, senna, ipecac and sulphate of soda are classed as hepatic stimulants—as undoubted cholagogues. Opium has a directly contrary effect.

Mercury, jaborandi and pellitory increase the flow of saliva. For the stimulation of the gastric secretion arsenic, alcohol, ipecac in small doses. bitters and alkalies before eating. are useful. Then the natural juice may be profitably supplemented by artificial means—pepsine and nitromuriatic acid are recommended. Sulphuric ether is unquestionably a pancreatic stimulant; and sulphate of magnesia largely increases the secretion of intestinal fluid.

We have thus attempted to give a brief, though very imperfect outline of the contents of this extremely entertaining, suggestive and instructive volume; and without committing ourselves unreservedly to every detail of the work, we heartily commend the book to our readers as eminently worthy, not only of attentive perusal but careful study.

---

A Manual of Histology. Edited and prepared by Thomas E. Satterthwaite, M.D., in association with Drs. Thomas Dwight, J. Collins Warren, William F. Whitney, Clarence J. Blake, C. H. Williams, J. Henry C. Simes, Benjamin F. Westbrook, Edmunnd C. Wendt, Abraham Mayer, R. W. Amidon, A. R. Robinson, W. R. Birdsall, D. Bryson Delavan, C. L. Dana and W. H. Porter. With one hundred and ninety-eight illustrations. Pages 478. New York: William Wood & Co. 1881.

The author has attempted, with the aid of his associates, to produce a book suitable for physicians and medical students. He has endeavored to avoid unnecessary diffuseness and wearisome detail on the one hand, and the brevity and incompleteness attaching to elementary treatises on the other.

It has been the aim of the writers to present the several subdivisions of the subject in a form as concise and simple as a due regard for perspicuity would admit, and in such a manner as to represent the most advanced state of knowledge in this important field of study.

For the purpose of securing these objects to the fullest extent, and in order to present the work as promptly as possible, the labor of its preparation was divided among a number of gentlemen, thought to be specially skilled in the department allotted to each. They have all discharged their trusts with admirable fidelity.

Many of the illustrations are new—all are good, and they do much toward simplifying the text.

A list of the necessary implements required for histological investigations is given. They are few, and not so expensive as is generally thought.

The book and all of the requisite apparatus can be purchased for a comparatively small outlay; and then the medical practitioner or student will be prepared to pursue inquiries in useful, interesting, and otherwise obscured paths of learning.

A practical knowledge of this subject should be possessed by every practitioner of medicine, not alone as tending to more exact habits of thought, and as productive of a broader culture, but also as a pleasant and profitable means of recreation and rest—rest from the humdrum routine of every-day life.

The appearance of the book is fresh and inviting. It contains a vast store of entertaining and practical knowledge; we can safely recommend it.

---

THE PRESCRIBERS MEMORANDA. Pages 301. New York: William Wood & Co. 1881.

This little book, from which the author has withheld his name—whether from modesty or timidity, does not appear—contains a large number of valuable prescriptions, selected, mainly, from the records of well-known practitioners, and also very brief, but admirable notes on the therapeusis of many diseases.

The custom of referring to ready-made formulæ in prescribing has been condemned, and perhaps justly, as calculated to encourage looseness in the selection and application of remedies, and as liable to beget a habit of routine, and consequently inexact and unscientific practice. This objection aside, we think the book before us is an excellent little work of its kind and likely to do good by suggesting the elegant, appropriate and effective combination of various drugs in order to meet certain given conditions.

---

ANTISEPTIC SURGERY The Principles, Modes of Application, and Results of the Lister Dressing. By Dr. Just Lucas—Championniere. Translated by Frederick Henry Gerrish, A.M., M.D. Portland, Me.: Loring, Short & Harmon. 1881.

This work, of 309 pages, treats elaborately of all the processes of antiseptic surgery, including corbolic spray and dressings, carbolized catgut ligatures, and drainage. Several chapters are devoted to its application to special operations, and the results in a number of cases presented.

While we do not agree entirely with all the statements of the enthusiastic author, we can recommend the book to those who desire to become conversant with all the principles and processes of Listerism.

THE WILDERNESS CURE. By Marc Cook. New York: Wm. Wood & Co.

This is the work of a non-professional, suffering with consumption, who having despaired of a cure by medicine, betook himself to camp life in the Adirondacks. His improvement was so marked that he felt justified in publishing the results for the benefit of others so affected. The book is written in a pleasant style; and the reader's sympathies and interest so secured that he must have heard with regret the announcement of the author's death sometime since, from the enemy that for a time seemed baffied.

---

FAVORITE PRESCRIPTIONS OF DISTINGUISHED PRACTITIONERS, WITH NOTES ON TREATMENT. Compiled by B. W. Palmer, A.M., M.D. New York: Birmingham & Co. 1881.

This is a little book of one hundred and twenty-one pages. Those who are fond of works of this character will find that the prescriptions are selected with rather more care than usual. A number of the most prominent physicians of the country are represented by these prescriptions. An index of diseases is appended for convenient reference to the remedies.

---

COULSON ON THE DISEASES OF THE BLADDER AND PROSTATE GLAND. Sixth Edition. Pp. 393. Revised by Walter J. Coulson, F.R.C.S. New York: Wm. Wood & Co. 1881.

This is the July volume of Wood's Library of Standard Authors, and is issued in the attractive form of that publication. The subject of which it treats is well presented No startling discoveries are announced, nor are any enticing new theories promulgated; but each chapter is well written, and the various plans of treatment carefully presented.

The chapter, in former editions, on the chemistry of urine, has been omitted in this one. After the anatomy and physiology of the bladder are given, a number of chapters are devoted to the injuries and diseases of that organ. A large part of the book is devoted to urinary concretions and their treatment. In the midst of this portion of the work we find injected, rather oddly, a chapter on foreign bodies in the bladder.

In connection with lithotrity, Bigelow's method, or litholopaxy, is described and very highly spoken of. In the preface, the author states that it "bids fair to create a a complete revolution in the doctrines hitherto current with regard to lithotrity, and in the mode of performing that operation."

Twenty-five pages are devoted to the diseases of the prostate gland. We fail to find here any mention of prostatorrhœa, as described in most works of this character, and especially dwelt upon by the elder and younger Gross.

The work will prove a useful and reliable guide to those interested in the subject.

